# Quantification of Total *Staphylococci* and *Escherichia coli* in Milk and Dairy Products from Small Ruminants and Characterization of the Antimicrobial Resistance Profiles of Isolated Pathogenic Strains

**DOI:** 10.3390/microorganisms13122756

**Published:** 2025-12-04

**Authors:** Sergiu Condor, Mihaela Duma, Smaranda Crăciun, Marian Mihaiu, Raluca Cîmpean, Oana Lucia Crisan-Reget, Sorin Daniel Dan, Laura Condor, Claudiu-Nicusor Ionica, Alexandra Tabaran

**Affiliations:** 1 Department of Animal Breeding and Food Safety, Faculty of Veterinary Medicine, University of Agriculture Sciences and Veterinary Medicine of Cluj-Napoca, 400684 Cluj-Napoca, Cluj County, Romania; sergiu.condor@usamvcluj.ro (S.C.); smaranda.craciun@student.usamvcluj.ro (S.C.); m.mihaiufmv@yahoo.com (M.M.); calina-raluca.cimpean@usamvcluj.ro (R.C.); oana.reget@usamvcluj.ro (O.L.C.-R.); sorindan@usamvcluj.ro (S.D.D.); claudiu-nicusor.ionica@usamvcluj.ro (C.-N.I.); 2 National Sanitary Veterinary and Food Safety Authority, 400621 Cluj-Napoca, Cluj County, Romania; duma.mihaelacj@yahoo.com; 3 National Sanitary Veterinary and Food Safety Authority, 510216 Alba-Iulia, Alba County, Romania; condor.laura-ab@ansvsa.ro

**Keywords:** *Staphylococcus aureus*, *Escherichia coli*, dairy products, antimicrobial resistance, virulence genes

## Abstract

This study evaluated the microbiological quality, presence of virulence genes, and antimicrobial resistance of *Staphylococcus aureus* and *Escherichia coli* in sheep and goat milk and traditional Romanian dairy products (Telemea and Burduf cheeses). Raw milk contained high levels of coagulase-positive staphylococci (CPS; 5.2 log CFU/mL) and *E. coli* (4.3 log CFU/mL), with several samples exceeding legal limits. Sour cream exhibited moderate CPS (1.2–1.9 log CFU/g) and *E. coli* (0.4–3.12 log CFU/g) counts, with occasional non-compliance. Cheeses had low CPS (0.52–0.84 log CFU/g) and *E. coli* (0.37–0.89 log CFU/g) levels, all within permissible limits. Molecular analysis detected the *nuc* gene in 21 sheep milk samples, of which 85.7% also carried the *sea* gene and 14.3% carried *seb*. Additionally, two goat milk samples tested positive for both *nuc* and *sea*. Three Telemea cheese samples were also *sea*-positive. Among raw milk samples, 10 *E. coli* isolates carried *stx1*, and two additionally harbored *hly*, while dairy products were negative for *E. coli* virulence genes. Antimicrobial susceptibility testing showed high resistance of *S. aureus* to penicillin (90.5%) and ampicillin (85.7%), with multidrug resistance among *sea*- and *seb*-positive isolates. STEC isolates showed resistance to ampicillin (70%), tetracycline (50%), and trimethoprim–sulfamethoxazole (40%), but remained susceptible to amoxicillin–clavulanic acid.

## 1. Introduction

Milk and dairy products from small ruminants such as sheep and goats are integral to Romania’s traditional and ecological agricultural heritage. These products are prized for their distinctive sensory attributes and nutritional value. However, their safety and hygienic quality can be compromised by microbial contamination, posing significant public health risks [1]. Among the microorganisms of concern, *Escherichia coli* and *Staphylococci*, particularly *Staphylococcus aureus*, are noteworthy due to their prevalence, pathogenic potential, and capacity to develop resistance to antimicrobial agents [2,3].

Among the different strains of *Escherichia coli*, certain types are pathogenic and pose significant health risks when present in dairy products. The most concerning are the Shiga toxin-producing *E. coli* (STEC), including the O157:H7 serotype, which can cause severe gastrointestinal illness, hemorrhagic colitis, and potentially life-threatening complications such as hemolytic uremic syndrome (HUS), especially in children and immunocompromised individuals [4,5]. The presence of *E. coli* in milk and dairy products is primarily indicative of fecal contamination, often resulting from inadequate hygiene during milking or improper handling during processing [6]. According to various European studies, the prevalence of pathogenic *E. coli* in raw milk and fresh dairy products from small ruminants can range from 2% to over 15%, depending on the region, production system, and season [7,8].

In Romania, a traditional product is legally defined as a food item created using local raw materials, without food additives, produced according to a traditional recipe, and obtained through a traditional method of preparation and/or processing that distinguishes it from other similar products. (Order no. 724/1.082/360/2013) [9]. Traditional dairy products, such as cheeses made from raw milk, are especially susceptible to contamination because they are not pasteurized, which allows pathogens to survive. Consequently, pathogenic *E. coli* poses a significant food safety risk, particularly in artisanal and small-scale production settings where hygiene measures may be variable or insufficient.

Conversely, *Staphylococcus* spp. are a common group of bacteria frequently detected in raw milk and dairy products, with their presence often linked to poor hygienic practices during milking, handling, or processing [10,11]. Among them, coagulase-positive *staphylococci* (CPS)—particularly *Staphylococcus aureus*—are of greatest concern due to their ability to produce heat-stable enterotoxins that are responsible for staphylococcal food poisoning, one of the most common foodborne intoxications worldwide [12]. While *S. aureus* can be present in low numbers in milk without causing harm, once bacterial loads exceed 10^3^ CFU/mL or CFU/g, especially under improper storage or fermentation conditions, there is a risk of toxin production [13]. These enterotoxins are resistant to heat and many standard food processing steps, meaning that even correctly ripened or heat-treated products may still pose a risk if contamination occurred early in the production chain. In Romania, several studies have highlighted the prevalence and antimicrobial resistance profiles of these pathogens in small ruminant milk and dairy products, but none of them has assessed the possible threat at a larger scale [14,15].

In raw sheep and goat milk—as well as in traditional Romanian dairy products such as cheese, telemea (a soft, salty cheese), sour cream, and burduf (a specialty cheese typically matured in a sheep’s stomach or casing)—Staphylococcus aureus has been detected at varying, yet frequently high, prevalence levels. Multiple studies conducted in Romania have reported the presence of *S. aureus* in 25–35% of raw milk samples from small ruminants, with many isolates carrying virulence determinants, including the *mecA* gene (associated with methicillin resistance) and various enterotoxin genes (*sea*, *seb*, *sec*, etc.) [16,17,18]. Traditional production practices—such as manual milking, the use of non-sterilized equipment, and the maturation of cheeses at temperatures above refrigeration—create conditions favorable to the growth of *S. aureus* and increase the risk of toxin formation, particularly when standardized hygiene measures are lacking [19].

Moreover, *S. aureus* is a frequent cause of subclinical mastitis in small ruminants, serving as a persistent reservoir of contamination. This contributes to high bacterial loads in milk even from apparently healthy animals. The risk is compounded in artisanal and small-scale farms, where veterinary supervision and routine microbial testing are often lacking [20]. Given the growing concern over antimicrobial resistance, *S. aureus* strains isolated from dairy products have also shown increasing levels of resistance to β-lactams, macrolides, tetracyclines, and aminoglycosides [20]. The emergence of methicillin-resistant *Staphylococcus aureus* (MRSA) in dairy herds not only complicates treatment but also presents a zoonotic risk to humans through direct contact or consumption of contaminated products [21].

The motivation for this study stemmed from the need to better understand the role of traditional Romanian dairy products made from small ruminant milk as potential reservoirs of pathogenic and antimicrobial-resistant bacteria, particularly *Escherichia coli* and *Staphylococcus aureus.* The specific objectives were to (i) determine the prevalence and counts of *E. coli* and *Staphylococci* in raw milk and traditional dairy products; (ii) identify pathogenic strains, including Shiga toxin-producing *E. coli* and coagulase-positive *S. aureus*; and (iii) evaluate their antimicrobial resistance profiles.

## 2. Materials and Methods

### 2.1. Sample Collection

Between April 2024 and June 2025, a total of 236 dairy samples were collected from various traditional markets located in the northwest region of Romania for microbiological determinations. The sample set included 126 individual raw milk samples—60 from goats and 66 from sheep—as well as 110 samples of dairy products, consisting of 30 samples of sour cream, 20 of telemea cheese (a traditional soft, salty cheese), 20 of fresh cheese (caș), and 20 of burduf cheese (a traditional Romanian cheese usually matured in sheep’s stomach or casing).

Samples were collected monthly throughout the study period to ensure seasonal variability was accounted for. The raw milk samples were obtained directly from producers or vendors selling unprocessed milk at traditional markets. Each milk sample was aseptically collected into sterile, food-grade screw-cap containers, with a minimum volume of 250 mL per sample. The milk was gently homogenized before collection to ensure sample consistency.

Dairy product samples were purchased in their original market packaging when available or were transferred aseptically using sterile spatulas and gloves into sterile, labeled containers. Approximately 200–250 g of each product was collected. All samples were labeled with the sample code, date of collection, type of product, market location, and animal origin (for milk samples).

Immediately after collection, the samples were placed in insulated coolers containing ice packs to maintain a temperature of 4 ± 2 °C. The cold chain was strictly maintained during transportation, and all samples were delivered to the laboratory within 4 to 6 h of collection. Upon arrival, the samples were either processed immediately or stored at 4 °C and analyzed within 24 h.

All microbiological determinations were performed using both biological and technical replication to ensure reliability. Each sample was processed as a single biological unit, and all culture-based assays were performed in technical duplicate (two independent plate inoculations per dilution). For each dairy product or milk sample, the primary homogenate (25 g or 25 mL in 225 mL buffered peptone) was prepared once and then used to produce at least two independent serial dilution series, from which duplicate plates were inoculated. Homogenization of solid samples was performed using a stomacher (2 min at ~230 rpm).

### 2.2. Determination of the Total Count of *Staphylococci* in Milk (Classical Method on Baird–Parker Agar)

The total count of *Staphylococci* in milk samples was determined using the classical culture method, based on the ISO 6888-1:2021 standard [22], which specifies the enumeration of coagulase-positive *Staphylococci* (particularly *Staphylococcus aureus*) by colony count technique following incubation on a selective medium.

Milk samples were first thoroughly homogenized, and serial tenfold dilutions were prepared in sterile peptone saline solution. From each dilution, 0.1 mL was aseptically inoculated onto Baird–Parker agar plates (Oxoid, London, England) supplemented with egg yolk tellurite emulsion. The inoculum was spread evenly over the surface using a sterile spreader to ensure uniform distribution.

For enumeration of *Staphylococci* on Baird–Parker agar, two plates were inoculated per dilution, and counts were recorded from plates within the 20–200 colony range. Plates were incubated aerobically at 37 ± 1 °C for 24–48 h. Following incubation, typical colonies of *Staphylococci* were identified by their characteristic appearance: black or grey, shiny, convex colonies with clear zones of precipitation (halo) around them due to lecithinase activity. Colonies exhibiting typical morphology were counted, and the number of *Staphylococci* was expressed as colony-forming units per milliliter (CFU/mL) of milk. The confirmation was done by Gram staining and a coagulase test (tube coagulase or latex agglutination) on one representative colony per distinct morphology. Sterility controls (uninoculated Baird–Parker plates) were included in each batch to monitor media sterility. A positive culture control (*S. aureus* reference strain, e.g., ATCC 25923) and a negative control (e.g., coagulase-negative *staphylococci* reference strain) were processed in parallel to validate selective performance.

### 2.3. Isolation and Enumeration of *Escherichia coli*

The enumeration of *Escherichia coli* in the milk and dairy products examined was performed using the selective culture method in accordance with ISO 16649-2 [23]. A representative sample (25 g from the dairy products and 25 mL from the milk samples) was homogenized in 225 mL of buffered peptone (Merck, Hamburg, Germany), followed by serial decimal dilutions in sterile diluent (peptone water).

Aliquots (1 mL) of appropriate dilutions were used for surface treatment on Tryptone Bile X-glucuronide (TBX) agar selective medium (Biolab Zrt, Budapest, Hungary) designed to detect β-glucuronidase-positive *E. coli*. Plates were incubated at 37 °C for 4 h for resuscitation, followed by an incubation at 44 ± 1 °C for 18–24 h. After incubation, the characteristic *E. coli* colonies (blue to blue–green) were selected for further analysis and counted.

*E. coli* enumeration on TBX agar was conducted with duplicate plates per dilution, just like in the case of *Staphylococcus* spp.; plates were incubated aerobically at 37 °C for 4 h for resuscitation, followed by 44 ± 1 °C for 18–24 h, as specified in ISO 16649-2 [2]. The confirmation was performed by oxidase testing (to exclude oxidase-positive contaminants) and biochemical confirmation (API 20E lent). Media sterility controls and a positive control strain (*E. coli* ATCC 25922 and, where available for virulence genes, an STEC control strain harboring *stx* genes) were included in each run.

The serotype of *E. coli* isolates was determined using conventional slide agglutination with commercially available antisera targeting O (somatic) and H (flagellar) antigens. Pure bacterial colonies were grown overnight on nutrient agar at 37 °C. A small amount of each colony was mixed with a drop of specific antisera on a clean glass slide and gently rocked for 1–2 min. Agglutination (clumping) was interpreted as a positive reaction, indicating the presence of the corresponding O or H antigen. Based on the agglutination pattern, isolates were assigned to the corresponding serotype.

### 2.4. PCR Protocol for Identification of Pathogenic Strains

#### 2.4.1. Colony Preparation and DNA Extraction

Colonies with typical *Staphylococcus* and *E. coli* morphology were selected for DNA extraction. The DNA extraction protocol was previously published by Mihaiu et al. (2014) [24]. Briefly, two to three specific colonies deriving from a single purified isolate were picked and suspended in 100 µL of sterile nuclease-free water or TE buffer. Afterward, the suspension was placed in a thermoblock (Bioline, London, UK) at 95 °C for 10 min to lyse the cells and release DNA. After boiling, the tube was centrifuged at 10,000 rpm for 5 min. The supernatant containing crude DNA was collected and used as a template for PCR.

#### 2.4.2. PCR Protocol

PCR amplification was performed in a 25 µL reaction volume containing 2.5 µL of 10× PCR buffer, 1.5–2.0 µL of MgCl_2_ (25 mM), 0.5 µL of dNTP mix (10 mM each), 1.0 µL each of forward and reverse primers (10 µM), 0.2 µL of Taq DNA polymerase (5 U/µL), 2–5 µL of DNA template, and nuclease-free water to adjust the final volume. Specific primers targeting major virulence genes of *E. coli* (*stx1*, *stx2*, *eaeA*, and *hlyA*) and *S. aureus* (*sea*, *seb*, *sec*, and *hla*) were used, based on previously published sequences [Table 1]. The thermal cycling conditions were as follows: an initial denaturation at 95 °C for 5 min, followed by 30 to 35 cycles of denaturation at 95 °C for 30 s, annealing at 55–60 °C for 30 s (depending on the primer set), and extension at 72 °C for 30 to 60 s. A final extension was performed at 72 °C for 5 min.

Amplified PCR products were analyzed by electrophoresis on 1.5% agarose gels stained with ethidium bromide and visualized under UV light. A 100 bp DNA ladder was used as a molecular size marker. Negative controls (PCR reaction without DNA template) were included in each run to monitor for contamination.

### 2.5. Antibiotic Susceptibility of the Isolated Pathogenic Strains

Antimicrobial susceptibility of *Staphylococcus aureus* and *Escherichia coli* isolates was evaluated using the classical disk-diffusion method on Mueller–Hinton agar, following the guidelines established by the Clinical and Laboratory Standards Institute [30]. After preparation of the standardized bacterial inoculum, antibiotic-impregnated disks representing different classes of antimicrobial agents were applied to the inoculated agar surfaces, and the plates were incubated under appropriate conditions to allow bacterial growth and antibiotic diffusion. For *S. aureus*, susceptibility testing included the following antibiotics: penicillin (10 U), ampicillin (10 µg), oxacillin (1 µg), erythromycin (15 µg), tetracycline (30 µg), gentamicin (10 µg), ciprofloxacin (5 µg), chloramphenicol (30 µg), and vancomycin (30 µg).

For *E. coli* isolates, the tested antibiotics comprised ampicillin (10 µg), amoxicillin–clavulanic acid (20/10 µg), ceftazidime (30 µg), cefotaxime (30 µg), gentamicin (10 µg), streptomycin (10 µg), tetracycline (30 µg), ciprofloxacin (5 µg), and trimethoprim–sulfamethoxazole (1.25/23.75 µg).

Following incubation, the diameters of the inhibition zones were measured and interpreted according to CLSI breakpoint criteria, and each isolate was categorized as susceptible, intermediate, or resistant to the tested agents. Reference control strains (*S. aureus* ATCC 25923 and *E. coli* ATCC 25922) were included to ensure quality control and reproducibility of the results.

### 2.6. Statistical Analysis

All statistical analyses were performed using Origin 8.5 (OriginLab Corporation, Northampton, MA, USA), applying one-way or two-way ANOVA followed by Tukey’s HSD post hoc test, with statistical significance set at *p* < 0.05.

## 3. Results

### 3.1. Microbiological Quality of Dairy Products

The microbial loads of coagulase-positive *Staphylococci* (CPS) and *Escherichia coli* were evaluated across four dairy products, including raw milk, sour cream, telemea cheese, and burduf cheese.

CPS and *E. coli* levels in raw milk averaged 5.2 log cfu/mL and 4.3 log cfu/mL, respectively. Several samples exceeded legal limits, indicating hygiene deficiencies at the farm or during initial handling. High CPS counts suggest a potential risk for staphylococcal foodborne intoxications, while elevated *E. coli* reflects fecal contamination and inadequate sanitary practices.

In the case of sour cream, the CPS values ranged from 1.2 and 1.9 lof cfu/g and the *E. coli* counts ranged from 0.4 to 3.12 log cfu/g. Two samples were non-compliant with legal standards in what concerns the *E. coli* number, suggesting post-processing contamination, likely during handling, packaging, or storage.

Both types of cheese (telemea, burduf) exhibited low CPS counts (0.52–0.81 log cfu/g in telemea; 0.6–0.84 log cfu/g in burduf) and *E. coli* levels (0.37–0.81 log cfu/g in telemea; 0.49–0.89 log cfu/g in burduf), remaining consistently within permissible limits (Table 2). These results indicate that the cheesemaking process, including fermentation, salting, and ripening, effectively reduces pathogenic bacterial loads, ensuring microbiological safety. Fresh cheese, which does not undergo fermentation or ripening, showed slightly higher CPS (0.8–1.3 log CFU/g) and *E. coli* levels (0.9–1.2 log CFU/g) than aged cheeses, but remained within permissible limits.

The statistical analysis revealed that CPS levels were highest in raw milk (5.2 ± 0.8 log CFU/mL) and significantly greater than in sour cream (1.6 ± 0.4 log CFU/g), telemea cheese (0.67 ± 0.12 log CFU/g), and burduf cheese (0.72 ± 0.15 log CFU/g) (*p* < 0.001, Tukey HSD). Similarly, *E. coli* counts were significantly elevated in raw milk (4.3 ± 0.6 log CFU/mL) compared to sour cream (1.76 ± 0.9 log CFU/g) and both cheeses (telemea: 0.59 ± 0.15; burduf: 0.69 ± 0.18 log CFU/g; *p* < 0.001). Pairwise comparisons showed no significant differences between telemea and burduf cheese for either CPS or *E. coli* (*p* > 0.05), suggesting that the cheesemaking process effectively reduced bacterial loads. Fresh cheese, although microbiologically safe, retains slightly higher bacterial loads due to the absence of these processing steps.

### 3.2. Molecular Analysis

Molecular analysis of the milk and dairy product samples revealed the presence of virulence-associated genes in both *Staphylococcus aureus* and *Escherichia coli* isolates.

### 3.3. Detection of Staphylococcus aureus Virulence Genes

Among the sheep milk samples, 21 were positive for the *nuc* gene, confirming the presence of *S. aureus.* Of these, 18 samples (85.7%) carried the *sea* gene, which was significantly higher than the 14.3% positive for *seb* (χ^2^ = 15.3, *p* < 0.001). None of the isolates harbored the sec gene.

In the goat milk samples, two were positive for the *nuc* gene, and both were also positive for the *sea* gene (Table 3).

From the dairy products tested, three Telemea cheese samples were found positive for the *sea* gene. Notably, these samples originated from the same producer whose milk samples also tested positive for *S. aureus* virulence genes.

*Detection of Escherichia coli virulence genes*

Among the 10 raw milk *E. coli* isolates carrying the *stx1* gene, molecular analysis and serotyping revealed that 5 isolates (50%) were O103, 2 isolates (20%) were O145, 2 isolates (20%) were O157:H7, and 1 isolate (10%) was O111. Two isolates also carried the *hly* gene, indicating enhanced virulence, and were found among the O103 (1) and O157:H7 (1) groups. No STEC virulence genes were detected in dairy product samples, suggesting that processing and cheesemaking effectively reduced the risk of pathogenic *E. coli*.

### 3.4. Antibiotic Susceptibility of the Pathogenic Strains

Of the 21 *S. aureus* isolates identified in sheep milk, the majority showed resistance to penicillin (19/21; 90.5%) and ampicillin (18/21; 85.7%). Intermediate prevalence was observed for erythromycin (12/21; 57.1%) and tetracycline (13/21; 61.9%), while reduced incidence levels were found for gentamicin (5/21; 23.8%), ciprofloxacin (6/21; 28.6%), and chloramphenicol (3/21; 14.3%). All isolates were susceptible to vancomycin (0/21; 0%).

Among sheep milk *S. aureus*, resistance to penicillin (90.5%) and ampicillin (85.7%) was significantly higher than resistance to gentamicin (23.8%) or chloramphenicol (14.3%; *p* < 0.01). *Sea*-positive isolates showed similar resistance profiles, while *seb*-positive isolates exhibited significantly higher resistance to more antibiotics, being resistant to penicillin, ampicillin, and tetracycline (χ^2^ = 6.8, *p* < 0.05).

Among the *sea*-positive isolates (n = 18), 16 (88.9%) were resistant to penicillin and 15 (83.3%) to ampicillin. Resistance to erythromycin and tetracycline was also frequent (10/18; 55.6% and 11/18; 61.1%, respectively).

The *seb*-positive isolates (n = 3) exhibited a slightly higher rate of multidrug resistance, being resistant to penicillin (3/3; 100%), ampicillin (3/3; 100%), and tetracycline (2/3; 66.7%).

The goat milk isolates (n = 2), both *sea*-positive, were resistant to penicillin and ampicillin, but remained susceptible to gentamicin and vancomycin. The Telemea cheese isolates (n = 3), also *sea*-positive, demonstrated a resistance pattern similar to the milk isolates, with resistance to penicillin (3/3; 100%) and ampicillin (3/3; 100%), and partial resistance to tetracycline (2/3; 66.7%).

Among the 10 *E. coli* isolates positive for the *stx1* gene, 7 (70%) were resistant to ampicillin, 5 (50%) to tetracycline, and 4 (40%) to trimethoprim–sulfamethoxazole. This resistance was significantly higher than resistance to gentamicin (20%) or chloramphenicol (20%; *p* < 0.05). Moderate resistance was also observed to ciprofloxacin (4/10; 40%), cefotaxime (3/10; 30%), and ceftazidime (3/10; 30%), suggesting potential extended-spectrum β-lactamase (ESBL) production. All isolates were susceptible to amoxicillin–clavulanic acid (0/10; 0%).

The two *hly*-positive isolates showed multidrug-resistant phenotypes, being resistant to ampicillin, tetracycline, ciprofloxacin, and cefotaxime simultaneously.

Overall, multidrug resistance (defined as resistance to three or more classes of antibiotics) was observed in 8/21 (38.09%) *S. aureus* isolates from sheep milk and in 7/10 (70%) *E. coli stx1*-positive isolates, with resistance most commonly detected to penicillin, ampicillin, and tetracycline (Table 4).

## 4. Discussion

The CPS and *E. coli* levels in Romanian dairy products that are produced in a traditional small-scale system are generally higher than those reported in previous articles. For instance, a study in Italy reported lower *E. coli* contamination in artisanal cheeses, attributed to stringent hygiene practices and controlled fermentation processes [17]. This underscores the need for improved hygiene and quality control measures in Romanian dairy production to align with international safety standards. The high values in some of the raw milk samples, both for CPS and *E. coli* levels, indicate significant microbial contamination, consistent with findings from other studies in Romania, where raw milk has been identified as a major source of pathogenic bacteria due to inadequate hygiene practices during milking and handling [16]. The fact that two samples of sour cream presented higher values for *E. coli* contamination suggests potential post-processing contamination, likely arising from handling or storage practices. Similar observations have been made in other studies, where dairy products like sour cream exhibited *E. coli* contamination linked to improper handling and storage conditions [31].

The presence of *S. aureus* and *E. coli* virulence genes in raw milk is a significant concern, as these pathogens can pose serious health risks to consumers. The detection of the *sea* and *seb* genes in *S. aureus* isolates indicates the potential for enterotoxin production, which can lead to foodborne illnesses. Similarly, the presence of the *stx1* and *hly* genes in *E. coli* isolates suggests the potential for severe gastrointestinal diseases.

These findings are consistent with other studies that have reported the presence of virulence genes in *S. aureus* and *E. coli* isolates from dairy products. For instance, a study by Ranjbar et al. (2018) [32] found that 25% of *E. coli* strains isolated from raw milk and traditional dairy products carried virulence genes, including *stx1* and *stx2*. Similarly, a study by Szczuka et al. (2022) [33] reported that 28% of *S. aureus* strains isolated from dairy products carried genes encoding enterotoxins, such as *sea* and *seb*.

Our findings revealed high resistance rates to commonly used antibiotics, notably penicillin, ampicillin, erythromycin, and tetracycline, among both *S. aureus* and *E. coli* isolates. These results are consistent with global trends observed in dairy-associated pathogens, underscoring the pervasive nature of AMR in dairy farming environments [30,31,32,33].

The high levels of β-lactam resistance observed among *S. aureus* isolates from sheep milk in this study (penicillin: 90.5%, ampicillin: 85.7%) are consistent with trends documented in earlier research. Lianou et al. (2021) [34], for example, reported penicillin resistance in 89,2% of *S. aureus* isolates from raw milk, underscoring the widespread presence of β-lactamase-producing strains in dairy environments. Similarly, Praja et al. (2023) [35] found penicilin resistance in 81,82% of isolates from dairy sheep in Indonesia. The moderate resistance detected to tetracycline (61.9%) and erythromycin (57.1%) also aligns with previous findings [31]. Tetracycline resistance in *S. aureus* from small ruminants is commonly reported at 25–46% [36,37], often linked to the presence of *tet* genes, while erythromycin resistance typically ranges from 30% to 60%, depending on geographic region and farm management practices. The relatively low resistance rates to gentamicin (8–28%), ciprofloxacin (28%), and chloramphenicol (4%), together with the universal susceptibility to vancomycin, likely reflect the limited use of aminoglycosides, fluoroquinolones, and glycopeptides in veterinary medicine, which has helped preserve their effectiveness against *S. aureus* in livestock [34,38].

The higher multidrug resistance observed in *seb*-positive isolates (80% resistance to penicillin and ampicillin, 41% to tetracycline) underscores the association between enterotoxin genes and resistance traits, consistent with studies suggesting that virulence and resistance genes can co-localize on mobile genetic elements [39]. *Sea*-positive isolates demonstrated slightly lower resistance rates, but still a notable frequency for penicillin and ampicillin, supporting the notion that enterotoxigenic *S. aureus* remains a significant concern in dairy products.

Both *sea*-positive isolates from goat milk and those derived from Telemea cheese exhibited uniform resistance to penicillin and ampicillin, reflecting the patterns observed in milk-derived strains. This underscores the potential for resistant *S. aureus* to be transmitted through dairy products. Comparable findings have been documented in raw goat milk and soft cheeses across Europe, where β-lactam resistance frequently exceeds 80% [40]. The partial tetracycline resistance observed in cheese isolates (66.7%) further suggests that resistance genes can persist within fermented dairy matrices.

Among *stx1*-positive *E. coli* isolates, 70% showed resistance to ampicillin, 50% to tetracycline, and 40% to trimethoprim–sulfamethoxazole, indicating a worrisome multidrug-resistant profile. Cephalosporin resistance (cefotaxime and ceftazidime) in 30% of isolates points to possible ESBL production, consistent with reports identifying ESBL-producing *E. coli* in small ruminants at prevalence rates of 20–35% [41,42]. Ciprofloxacin resistance in 40% of isolates is higher than the <30% generally reported in European studies, although it aligns with resistance trends observed in regions where fluoroquinolones are extensively used in livestock.

The *hly*-positive *E. coli* isolates exhibited multidrug resistance, paralleling previous observations that hemolysin-producing strains often carry multiple resistance determinants [43]. Importantly, all *E. coli* isolates remained susceptible to amoxicillin–clavulanic acid, suggesting that β-lactamase inhibitors remain effective treatment options.

The co-occurrence of virulence and resistance genes in both *S. aureus* and *E. coli* likely reflects the action of mobile genetic elements, which facilitate horizontal gene transfer within the microbial communities of the udder and milk environment [44,45]. Biofilm formation on teat surfaces, milking equipment, or dairy matrices can further promote persistence and exchange of these elements, while suboptimal hygiene allows for higher bacterial loads, increasing the probability of gene transfer. The absence of virulence genes in processed dairy products suggests that fermentation, salting, and ripening reduce viable pathogen numbers and may impair the maintenance of mobile genetic elements, providing a natural barrier against the dissemination of pathogenic traits.

The multidrug-resistant phenotypes observed in *stx1*- and *hly*-positive *E. coli* isolates may be mechanistically linked to co-selection pressures, where exposure to one antimicrobial class maintains resistance to others through linked genes on plasmids [46]. Hemolysin and Shiga toxin production can also confer a fitness advantage during colonization, promoting persistence in the udder or gut despite antimicrobial interventions. These mechanisms underscore the importance of combined strategies targeting both hygiene and antimicrobial stewardship to limit the spread of virulent, resistant strains.

Finally, the persistence of resistant and virulent *S. aureus* in Telemea cheese demonstrates that artisanal processing alone is insufficient to eliminate high-risk strains, particularly when raw milk from contaminated animals is used. The use of controlled starter cultures, proper acidification, and extended ripening at appropriate temperatures could further reduce pathogen survival and mitigate food safety risks [17,46].

While our study provides valuable insights into the prevalence of *S. aureus* and *E. coli* and their multidrug resistance profiles in small-scale Romanian dairy products, further investigations are warranted to assess the presence of extended-spectrum β-lactamases (ESBLs) and carbapenemase-producing isolates, which would offer a more comprehensive understanding of antimicrobial resistance trends and their public health implications.

## 5. Conclusions

This study provides a comprehensive assessment of the microbiological quality, virulence factors, and antimicrobial resistance patterns of *Staphylococcus aureus* and *Escherichia coli* isolated from traditionally processed milk and dairy products derived from small ruminants in Romania. The elevated bacterial loads observed in raw milk samples indicate notable hygiene deficiencies during milking and initial handling. In contrast, fermented products such as Telemea and Burduf cheese exhibited satisfactory microbiological safety, highlighting the effectiveness of fermentation and ripening processes in reducing pathogenic microorganisms.

The detection of virulence-associated genes—*sea* and *seb* in *S. aureus* and *stx1* and *hly* in *E. coli*—underscores the potential health risks posed by these pathogens in unprocessed milk. The recurrence of identical virulence profiles in both milk and cheese from the same producers suggests possible cross-contamination along the production chain.

Antimicrobial susceptibility testing revealed widespread resistance to β-lactams among *S. aureus* isolates and multidrug resistance among *E. coli*, with some evidence of extended-spectrum β-lactamase activity. Despite these findings, susceptibility to gentamicin, vancomycin, and amoxicillin–clavulanic acid was retained.

Overall, the study highlights the need for stringent hygiene management, systematic microbial and molecular monitoring, and prudent antimicrobial use in dairy farming. Implementing these measures is crucial to mitigating the spread of resistant and virulent bacterial strains, ensuring consumer safety and compliance with international dairy quality standards.

## Figures and Tables

**Table 1:** Primer sequences used for PCR detection of *Staphylococcus aureus* and *E. coli* virulence genes.

Organism/Target Gene	Primer Sequence	Amplicon Size (bp)	Reference
*Staphylococcus aureus*/*nuc*	F:TACAAAGGTCAACCAATGACATTCAGACTA R:TAAATGCACTTGCTTCAGGGCCATAT	687	Wolk et al., 2009 [25]
*S. aureus*/*sea*	GGTTATCAATGTGCGGGTGG CGGCACTTTTTTCTCTTCGG	102	Becker et al., 1998 [26]
*S. aureus*/*seb*	GTATGGTGGTGTAACTGAGC CCAAATAGTGACGAGTTAGG	164	Becker et al., 1998 [26]
*S. aureus*/*sec*	AGATGAAGTAGTTGATGTGTATGG CACACTTTTAGAATCAACCG	451	Becker et al., 1998 [26]
*E. coli*/*Stx1*	F:GACTGCAAAGACGTATGTAGATTCG R:ATCTATCCCTCTGACATCAACTGC	150	Hein et al., 2001 [27]
*E. coli*/*Stx2*	ATTAACCACACCCCACCG GTCATGGAAACCGTTGT CAC	200	Ibekwe et al., 2002 [28]
*E. coli*/*eae*	GTAAGTTACACTATAAAGCACCGTCG TCTGTGTGGATGGT AATAAATTTTTG	106	Ibekwe et al., 2002 [28]
*E. coli*—*hlyA*	ACGATGTGGTTTATTCTGGA CTTCACGTGACCATACATAT	165	Fagan et al., 1999 [29]

**Table 2:** Incidence and microbial loads of CPS and *E. coli* in dairy products.

Dairy Product	No. of Samples	CPS (log CFU/g or mL)	*E. coli* (log CFU/g or mL)	Samples Exceeding Legal Limits
Raw milk	126	5.2 ± 0.8 log CFU/mL	4.3 ± 0.6 log CFU/mL	Yes (several)
Sour cream	30	1.2–1.9 log CFU/g	0.4–3.12 log CFU/g	2 (*E. coli*)
Telemea cheese	20	0.52–0.81 log CFU/g	0.37–0.81 log CFU/g	No
Fresh cheese	20	0.8–1.3 log CFU/g	0.9–1.2 log CFU/g	No
Burduf cheese	20	0.6–0.84 log CFU/g	0.49–0.89 log CFU/g	No

**Table 3:** Detection of virulence genes and resistance profiles in *S. aureus* and *E. coli*.

Species	Source	No of Positive Samples	Virulence Genes Detected Serotype (*E. coli*)	Resistance Profile/No of Samples
*S. aureus*	Sheep milk	21	*nuc* (100%), *sea* (85.7%), *seb* (14.3%), *sec* (0%)	PEN, AMP, ERY, TET/8 PEN, AMP, TET/13
*S. aureus*	Goat milk	2	*nuc* (100%), *sea* (100%)	PEN, AMP/2
*S. aureus*	Telemea cheese	3	*sea* (100%)	PEN, AMP, TET/2 PEN, AMP/1
*S. aureus*	Fresh cheese	2	*sea* (100%)	PEN, AMP/1 PEN, AMP, ERY, TET/1
*E. coli*	Raw milk	10	*stx1* (10/10), *hly* (2/10) O103: 5 isolates (50%), O111: 1 (10%), O145: 2 (20%), O157:H7: 2 (20%)	AMP, TET, SXT/4 AMP, TET, SXT, CIP/3

**Table 4:** Antimicrobial resistance patterns in *S. aureus* and *E. coli* isolates.

Species	Source	Virulence Gene	No. of Isolates	Resistance Profile
*S. aureus*	Sheep milk	*Sea*	8	PEN, AMP, ERY, TET
*S. aureus*	Sheep milk	*Sea*	10	PEN, AMP, TET
*S. aureus*	Sheep milk	*Seb*	3	PEN, AMP, TET
*S. aureus*	Goat milk	*Sea*	2	PEN, AMP
*S. aureus*	Telemea cheese	*Sea*	2	PEN, AMP, TET
*S. aureus*	Telemea cheese	*Sea*	1	PEN, AMP
*E. coli*	Raw milk	*Stx1*	4	AMP, TET, SXT
*E. coli*	Raw milk	*Stx1*	3	AMP, TET, SXT, CIP

PEN = penicillin; AMP = ampicillin; TET = tetracycline; ERY = erythromycin; CIP = ciprofloxacin; SXT = trimethoprim–sulfamethoxazole; CTX = cefotaxime.

## Data Availability

The data presented in this study are available on request from the corresponding author due to (protect the privacy of the participants).
